# A modified shuffled frog leaping algorithm with inertia weight

**DOI:** 10.1038/s41598-024-51306-1

**Published:** 2024-02-20

**Authors:** Zhuanzhe Zhao, Mengxian Wang, Yongming Liu, Yu Chen, Kang He, Zhibo Liu

**Affiliations:** 1 School of Mechanical Engineering, Anhui Polytechnic University, Wuhu, Anhui China; 2 School of Artificial Intelligence, Anhui Polytechnic University, Wuhu, Anhui China; 3 School of Mechanical and Electronic Engineering, Suzhou University, Suzhou, Anhui China

**Keywords:** Computational biology and bioinformatics, Mathematics and computing

## Abstract

The shuffled frog leaping algorithm (SFLA) is a promising metaheuristic bionics algorithm, which has been designed by the shuffled complex evolution and the particle swarm optimization (PSO) framework. However, it is easily trapped into local optimum and has the low optimization accuracy when it is used to optimize complex engineering problems. To overcome the shortcomings, a novel modified shuffled frog leaping algorithm (MSFLA) with inertia weight is proposed in this paper. To extend the scope of the direction and length of the updated worst frog (vector) of the original SFLA, the inertia weight α was introduced and its meaning and range of the new parameters are fully explained. Then the convergence of the MSFLA is deeply analyzed and proved theoretically by a new dynamic equation formed by Z-transform. Finally, we have compared the solution of the 7 benchmark functions with the original SFLA, other improved SFLAs, genetic algorithm, PSO, artificial bee colony algorithm, and the grasshopper optimization algorithm with invasive weed optimization. The testing results showed that the modified algorithms can effectively improve the solution accuracy and convergence property, and exhibited an excellent ability of global optimization in high-dimensional space and complex function problems.

## Introduction

Optimization problem refers to the search for optimal solutions to some practical problems in the process of human production and life under a set of constraints. Meta-heuristic algorithm is one of the best methods to deal with this kind of problem^1^. It is simple and flexible in computation, and its optimization scope is not only suitable for specific fields, but also has no special requirements for objective function. In recent years, with the development of meta-heuristic optimization algorithms, many complex optimization problems can be solved easily and effectively, and natural meta-heuristic algorithms have become a research hot spot^2,3^. Most natural meta-heuristics are inspired by the behavior or physical phenomena of groups of organisms in nature. For example, the whale optimization algorithm (WOA)^4^ simulates humpback whale's unique search method and rounding mechanism, which mainly includes three important stages: rounding up prey, bubble net hunting, and searching prey. The marine Predators Algorithm (MPA)^5^ was inspired by marine predators' survival of the fittest theory, that is, marine predators chose the best foraging strategies by choosing between Levy and Brownian movement. The dragonfly algorithm (DA)^6^ is mainly inspired by the static and dynamic group behavior of dragonflies in nature. The moth flame optimization algorithm (MFO)^7^ is inspired by the navigation mechanism of moths chasing flames in the direction of lateral flight. Atomic orbital search (AOS)^8^ is inspired by some of the principles of quantum mechanics and quantum-based models of the atom, taking into account the properties of electrons around the nucleus. The gazelle optimization algorithm (GOA)^9^ mainly simulates the behavior of antelopes escaping predators. Many natural meta-heuristic algorithms have excellent performance that is efficient, simple and avoids falling into locally optimal. However, the No Free lunch theory has stated that no algorithm can solve all optimization problems, nor can it perform well in all problems. Therefore, continuous improvement of algorithms is crucial to solve more practical optimization problems.

The shuffled frog leaping algorithm (SFLA) has been known as a metaheuristic population-based algorithm which was originally introduced by Eusuff and Lansey^10^. This algorithm was motivated by the predatory habit of frog groups in a small pond and contains elements of local search and global information shuffling^10,11^. Due to its advantages of fast computation and excellent convergence performance, SFLA has been widely applied in optimization domains, such as parameter estimation^12^, the unit commitment problem^13^, wireless sensor networks (WSNs) design^14^, integrated circuits design^15^, scheduling problem^16^, and machine learning^17^. However, with the increasing complexity and the dimension of the solving problem, the convergence speed and solution accuracy of SFLA decreases significantly, even the SFLA easily traps into local optima. Thus, researchers conducted various improvements on SFLA to develop new algorithms for the improvements in its performance. Four versions of SFLA were proposed by using the opposition-based learning (OBL) strategy in the SFLA to diversify the search moves and accelerate search process^18^. A new differential operator was inserted in the evolutionary process of the SFLA to prevent a premature loss of genotypic diversity. An adaptive frog leaping rule based on the genetic mutation operator was suggested to enhance the local exploration and performance of the initial SFLA^19^. A combination of non-local spatial information and quantum-inspired SFLA^20^ and a hybrid SFLA with antipredator capabilities to avoid the local minima^21^ have been proposed. A novel scheme based on quantum evolution strategy and eigenvector evolution strategy was introduced. In this scheme, the frog leaping rule based on quantum evolution is achieved by two potential wells with the historical information for the local search, and eigenvector evolution is achieved by the eigenvector evolutionary operator for the global search^12^. By introducing acceleration factors *c*_1_ and *c*_2_ into the basic SFLA^22^, the ability of the worst individual to learn from best individual within the sub-memeplexes or global best individual of the entire population was improved and the convergence rate of algorithm was accelerated. Meanwhile, some novel hybrid SFLA exhibited integrations in other intelligence algorithms, such as the genetic algorithm (GA)^19^, simulated annealing (SA)^23^, harmony search (HS)^24^, particle swarm optimization (PSO)^25^, which have been greatly advanced the hybridizing work of SFLA algorithms. Technically speaking, these improved algorithms or their variants can improve the SFLA’s performance, such as faster convergence speed, higher accuracy of solution, increased local exploration ability and so on. However, with more and more complex practical optimization problems and strict real-time requirements, it is necessary to find the SFLA with the relatively small computational complexity, the higher solution accuracy and better global optimization performance. Therefore, there is still much room for the improvement of the original SFLA.

Animals have an instinctive ability to remember their past actions (for example, the path they have traveled, or an action). In the next similar activity, appropriate adjustments and changes will be made based on the previous behavior, rather than a complete restart. This kind of behavior is called inertial behavior, which is the inheritance and reference of past experience, and helps animals quickly achieve their own purpose. However, the current improved SFLA still has some shortcomings, such as too many improvement points but the effect is not obvious, the content is complex, the relevant papers lack mathematical theory. On this basis, introducing the inertia weight parameter into the meme evolution strategy not only effectively enhances the performance of the original algorithm, but also the concepts involved in MSFLA are relatively simple, easy to understand and flexible. In this paper, the convergence of the algorithm is analyzed theoretically and the value range of the inertia weight parameter is given, which provides a theoretical basis for the related research of SFLA. Moreover, compared with the original SFLA algorithm, the computation cost and time complexity are not increased, and the operation is more convenient.

The remainder of the paper is organized as follows. The original SFLA is briefly described in Sect. "Original shuffled frog leaping algorithm". In Sect. "Modified shuffled frog leaping algorithm (MSFLA)", "Inertia weight strategies of MSFLA", the MSFLAs with three different inertia weight strategies are presented to extend the scope of the direction and length of the worst frog, where the superiority of MSFLA over original SFLA is demonstrated by the vector syntheses on 2-dimensional space. The reasonable range of new parameter was discussed in mathematical theory, and it was listed in detail. Three modified SFLA models and the original SFLA are applied for the seven typical benchmark test problems, as shown in Sect. "Experiment and discussions". Moreover, the simulation results demonstrate the effectiveness of the modified algorithms. Section "Engineering design problems" is the result of the improved algorithm in the engineering optimization case, and Sect. "Conclusions" summarizes this paper.

## Original shuffled frog leaping algorithm

**Figure 1:**
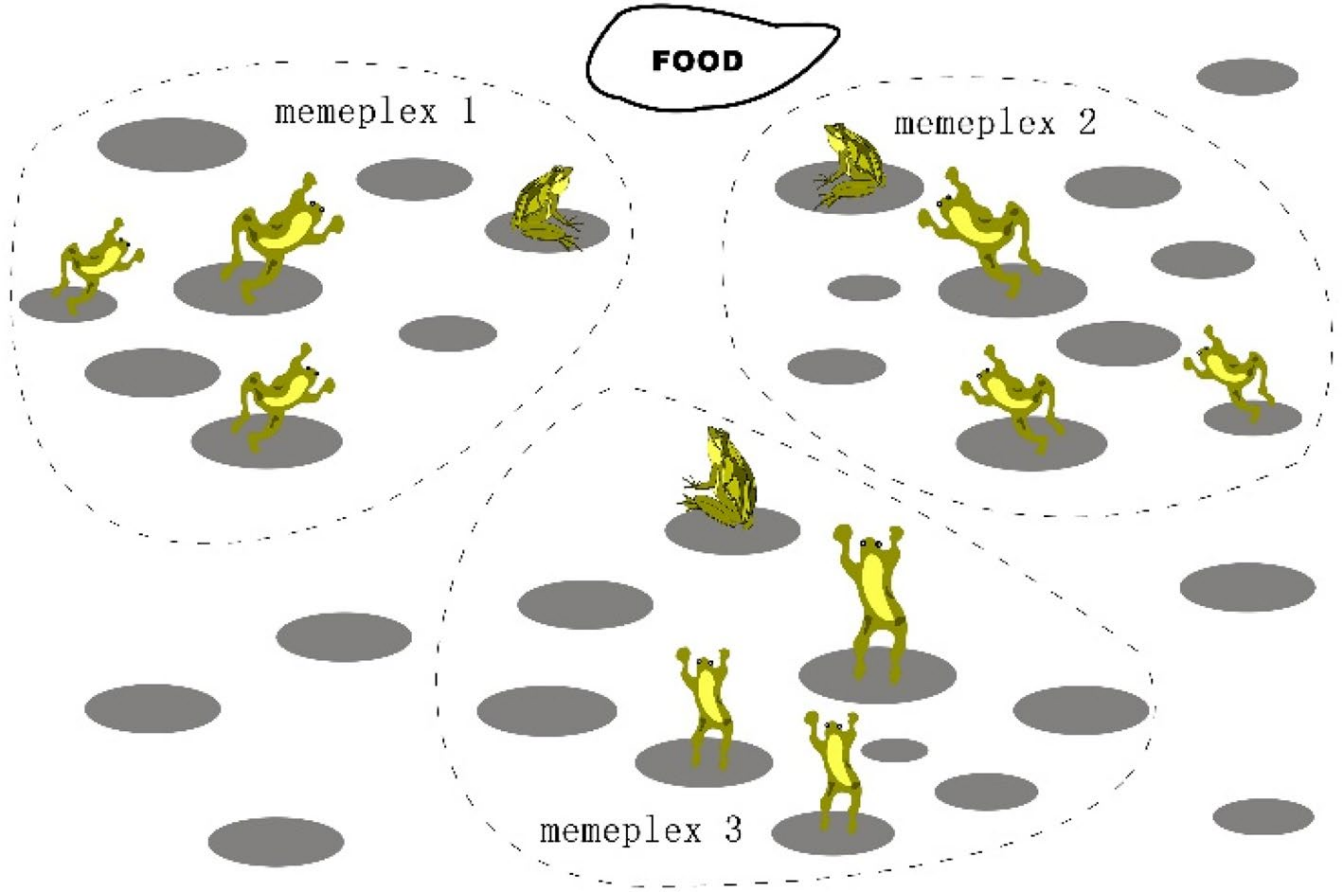
The basic concept of SFLA.

A frog population lives in a swamp or pond, and there are many discrete stones for frogs to jump when looking for food. Frog individuals are allowed to communicate with each other, so as to learn from the experience of other individuals to improve their own jumping direction and step size, and achieve the purpose of information sharing. In order to find food quickly and accurately, the frog population is divided into several memeplexes with the same number but different abilities to form a small group in a local range. The local elite individuals guide other individuals to search for food independently in different directions. After each memeplex has searched a certain number of times, different memeplex exchange information through each memeplex shuffling, which makes many frogs learn the new ideas of different memeplexes and realize the social sharing of information so that the whole frog population can quickly and successfully find the food source in the right direction. The basic concept of the SFLA is shown in Fig. 1.

The original SFLA is a combination of random and deterministic approaches. The deterministic strategy, the local and global explorations, could effectively ensure evolution guide of the algorithm toward the global optimum using the heuristic information (or fitness function). The random elements also could improve the flexibility and robustness of search pattern. Some main steps of the algorithm are shown below^10,11^.

*Step 1* A virtual population of *F* different frogs is generated randomly in the feasible *D*-dimensional space. Each frog represents a candidate solution of optimization problem and *D* is the number of decision variables. So the ${i}_{th}$ frog is expressed by a vector ${U}_{i}=\left({U}_{i1},{U}_{i2},...,{U}_{iD}\right)$. Each frog has an associated fitness value ${f}_{i}$ that measures the performance of the frog.

*Step 2* All frogs are sorted in a descending order according to their fitness values and the entire population is partitioned into *m* memeplexes (communities)*Y*^1^,*Y*^2^,⋯${Y}^{m}$, each containing *n* frogs (i.e. *F* = *m* × *n*), such that

1 $$\mathop Y\nolimits^{k} = \left[ {U_{j}^{k} ,f_{j}^{k} \left| {U_{j}^{k} = U_{k + m(j - 1)} ,\;f_{j}^{k} = f_{k + m(j - 1)} ,\;j = 1, \cdots ,n} \right.} \right],\quad k = 1, \cdots ,m$$

Record the frog with the best fitness value as ${U}_{g}$ in the entire population.

*Step 3* The memetic evolution of SFLA starts. Firstly, *q* distinct frogs are selected randomly from *n* frogs within the memeplex ${Y}^{m}$ to construct a submemeplex. The selection strategy is to give a higher probability of being selected to the frogs that have higher performance values. The frogs within submemeplex are resorted in order of decreasing performance. For each submemeplex, the frogs with the worst and the best performance are identified as ${U}_{w}$ and ${U}_{b}$, respectively. Then, the worst frog ${U}_{w}$ in each submemeplex is updated as follows:

2 $$S = \left\{ {\begin{array}{*{20}c} {{\text{min}}\left[ {r\left( {U_{b} - U_{w} } \right),S_{\max } } \right],} & {U_{b} - U_{w} \ge 0} \\ {{\text{max}}\left[ {r\left( {U_{b} - U_{w} } \right), - S_{\max } } \right],} & {U_{b} - U_{w} < 0} \\ \end{array} } \right.$$

where *S* is the updated step size and is a *D*-dimensional vector; *r* is a random number between 0 and 1; ${S}_{max}$ is the maximum step size allowed to be adopted by a frog after being infected. The new frog is then computed by

3 $$U_{w}^{\prime} = U_{w} + S$$

**Figure 2:**
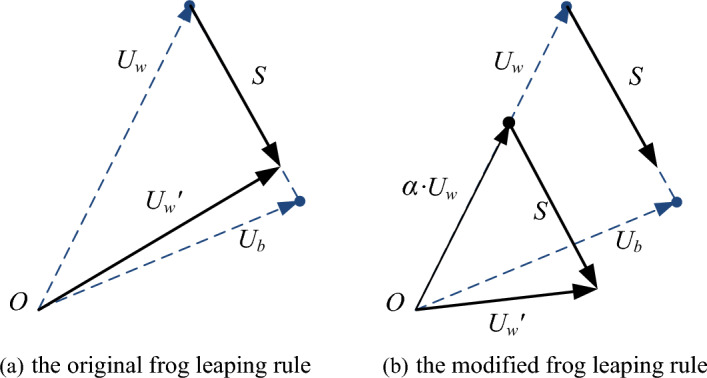
The vector syntheses on 2- dimensional space.

The evolution rule presented above is shown as Fig. 2a.

If the performance of the new ${U}_{w}^{,}$ is better than the old ${U}_{w}$, it replaces the worst ${U}_{w}$. Otherwise, the calculations in Eqs. (2) and (3) are repeated with respect to the global best frog, i.e., ${U}_{b}$ is replaced by ${U}_{g}$. If no improvement becomes possible in this case, then a new frog (solution) is randomly generated to replace the frog ${U}_{w}$. This operation is repeated by the required number of iterations ${L}_{max}$. The search process above is called the local exploration of the SFLA.

*Step 4* Once the local exploration is completed for the *m* memeplexes, the algorithm returns to the global exploration for shuffling. For a global information exchange, the frog population is rearranged in accordance with the new fitness values. Update the global best frog ${U}_{g}$. Then, the entire frogs are partitioned into *m* memeplexes and a new local search starts again. The local exploration and global shuffling process are carried out alternatively until the iteration numbers ${G}_{max}$ or convergence criteria are satisfied. The updated ${U}_{g}$ is the optimal solution of optimization problem.

The main parameters of the SFLA are: number of frogs *F*, number of memeplexes *m*, number of frogs in each memeplex *n*, number of frogs in each submemeplex *q* and the maximum local search number of evolutionary iterations ${L}_{max}$ before shuffling. The last parameter is the stop criteria of algorithm. It can be the maximum iterations number of global shuffling ${G}_{max}$ or the solution accuracy *ɛ*.

## Modified shuffled frog leaping algorithm (MSFLA)

The original frog leaping rule is inspired by this natural memetics (see Fig. 2a). As can be seen from the figure, the possible position of the updated new frog ${U}_{w}^{,}$ is restricted in the narrow area between its old position and the best frog’s position ${U}_{b}$ (or ${U}_{g}$), and its length and direction will never surpass the best one. Therefore, it indicates that the performance of ${U}_{w}^{,}$ is not better than the performance of ${U}_{g}$ in the process of evolution^26^.

Clearly, this frog leaping rule limits the local search space in the memetic evolution process and might fall into the local optimum. To overcome this limitation, a modified frog leaping rule is introduced in this study. From the perspective of social cooperation, the second part of Eq. (3) represents its social ability to learning from others. Meanwhile, the first part represents the ability to self-diagnose in the evolution step. In SFLA, the evolution process is only applied to update the frog with the worst performance (i.e. not all frogs) within each submemeplex, which is obviously different from the other swarm intelligence algorithms. Therefore, in the ideal case, the updated new frog should inherit and increase the advantages of the better one, while reducing the impact of the old worst frog as far as possible. On the basis of the analysis mentioned above, a new parameter called the inertia weight *α* is introduced to improve the original frog leaping rule by controlling the inherited share from the worst frog. The new frog leaping rule is expressed as:

4 $$U_{w}^{\prime} = \alpha U_{w} + S$$

This new parameter *α* displays roles in balancing the self-cognitive ability and team learning capability of the worst frog. Besides, the new parameter *α* can not only make the worst frog maintain the leaping inertia, but it also greatly increases the diversity of the solution. If *α* = 0, the worst frog has no self-cognitive ability and the algorithm would be trapped into the complete random state. If *α* > 1, the newly updated frog would keep the much gene of the worse performance and the convergence speed will slow down greatly. When *α* = 1, it is the same as Eq. (3). So the reasonable range of inertia weight *α* is in the range 0–1. The 2-dimensional vector syntheses of the modified frog leaping rule is demonstrated in Fig. 2b. It can be seen that the new rule can extend the direction and the length of each frog’s jump. Through widening the local search space, the MSFLA helps to prevent premature convergence and effectively improve the solution performance.

Theoretically, the inertia weight can be a positive constant or even a positive linear or nonlinear function of time. If *α* is a constant, especially set as an unreasonable value, the diversity of MSFLA could decrease. Thus, it is contrary to the original improved intention. Therefore, in this research, the three time-varying strategies for determining the value of inertia weight are proposed and form the different modified models of SFLA, which are inspired by the inertia weight strategies of the PSO.

To better analyze, assuming the number of frog memeplex is *m* = 1, then the ${U}_{b}$=${U}_{g}$. At the same time, assuming the maximum step size ${S}_{max}$ of frog-leaping can be allowed infinite as long as it does not exceed the domain of definition. Then the updating formula of the worst frog MSFLA (Eq. (4)) and Eq. (2) can be combined and simplified to the following form

5 $$U_{w} (k + 1) = \alpha U_{w} (k) + r[U_{b} \left( k \right) - U_{w} \left( k \right)]$$

where *k* is the iteration number of global search. We can obtain Eq. (6) by simplifying the Eq. (5):

6 $$U_{w} \left( {k + {1}} \right){ - [}\alpha { - }r{]}U_{w} \left( k \right) = rU_{b} \left( k \right)$$

Suppose the MSFLA is convergent, then with the iteration number *k* increasing, ${U}_{w}(k)$ and ${U}_{b}(k)$ are formed as two discrete time sequences with global convergence. Now their *z*-transform exist and can be noted as ${U}_{w}(z)$ and ${U}_{b}(z)$. Perform *z*-transform onto both sides of Eq. 6 under zero initial condition.

7 $$zU_{w} \left( z \right){ - [}\alpha { - }r{]}U_{w} \left( z \right) = rU_{b} \left( z \right)$$

Therefore, the system (MSFLA) described by Eq. (7) can be considered as a discrete time dynamic causal system whose reference input is ${U}_{b}(z)$ and system output is ${U}_{w}(z)$. Therefore, the system transfer function is shown below

8 $$H\left( z \right) = \frac{{U_{w} \left( z \right)}}{{U_{b} \left( z \right)}} = \frac{r}{z - (a - r)}$$

And the precondition of the system convergence is that the system must be stable. The necessary and sufficient condition of system stability is that the poles of *H*(*z*) are all in the unit circle. That is satisfied with the following condition:

9 $$z = \left| {\alpha - r} \right| < 1$$

For $0 < \alpha < 1$, the inequality (9) is clearly established. That is because when $0 < r < 1$, the inequality $0 < \left| {\alpha - r} \right| < 1$ is satisfied. So the original hypothesis is established, that is, MSFLA must be convergent.

For $1 \le \alpha < 2$, the inequality (9) has the possibility of existence, that is, there is the possibility of convergence of MSFLA, but this will add a number of unstable factors to the stability of MSFLA; But for $\alpha \ge 2$, the inequality $\left| {\alpha - r} \right| > 1$ is satisfied and the system *H*(*z*) is unstable. it means that the MSFLA will be no longer converge.

## Inertia weight strategies of MSFLA

### Random inertia weight

In the solution process of the actual question, the required value of inertia weight could be different in each memetic generation. Usually, *α* can come from a certain function distribution, such as the uniform distribution, random distribution, and normal distribution. A random value of inertia weight is used to enable the MSFLA to track the global optima. The formula is as follows^27^:

10 $$\alpha = 0.5 + {r \mathord{\left/ {\vphantom {r 2}} \right. \kern-0pt} 2}$$

where *r* is a random number in [0, 1] and it is the same in Eq. (2); *α* is then a uniform random variable in the range [0.5, 1]. The modified SFLA model with the random inertia weight strategy is denoted as the MSFLA-R.

### Linear time-varying inertia weight

In most of the PSO variants, the inertia weight value is determined by the iteration number, which is called the time-varying inertia weight strategy. A linear decreasing time-varying inertia weight was first introduced in Shi’s and Eberhart’s studies^28^ and experimental results show that the strategy is an effective approach. In view of this, the same strategy of inertia weight is applied to the MSFLA model according to the following equation:

11 $$\alpha \left( {iter} \right) = \alpha_{\max } - \left( {\alpha_{\max } - \alpha_{\min } } \right){{iter} \mathord{\left/ {\vphantom {{iter} {L_{\max } }}} \right. \kern-0pt} {L_{\max } }}$$

where $iter$ is the current iteration of local exploration within each memeplex; ${\alpha }_{max}$ and ${\alpha }_{min}$ are the maximum value and the minimum value of the inertia weight *α*. In this method, the inertia weight value is linearly decreased from the initial value (${\alpha }_{max}$) to the final value (${\alpha }_{min}$) according to the local iteration number within each memeplex. The modified SFLA model with the linear time-varying inertia weight strategy is denoted as the MSFLA-L.

### Nonlinear time-varying inertia weight

The memetic evolution (or search) process is very complex and nonlinear in most intelligent algorithms. Some researchers proposed nonlinear adjustment strategies of inertia weight in the PSO variants. A typical nonlinear strategy of inertia weight is used in the MSFLA model as the following quadratic function^29^:

12 $$\alpha \left( {iter} \right) = \left( {\alpha_{1} - \alpha_{2} } \right)\left( {\frac{{iter - L_{\max } }}{{L_{\max } }}} \right){}^{2} + \alpha_{2}$$

where *α*_1_ and *α*_2_ are the initial and final values of inertia weight. In each local exploration process, the inertia weight starts from *α*_1_ and ends at *α*_2_. The modified SFLA model with the quadratic weight strategy is denoted as the MSFLA-Q.

**Figure 3:**
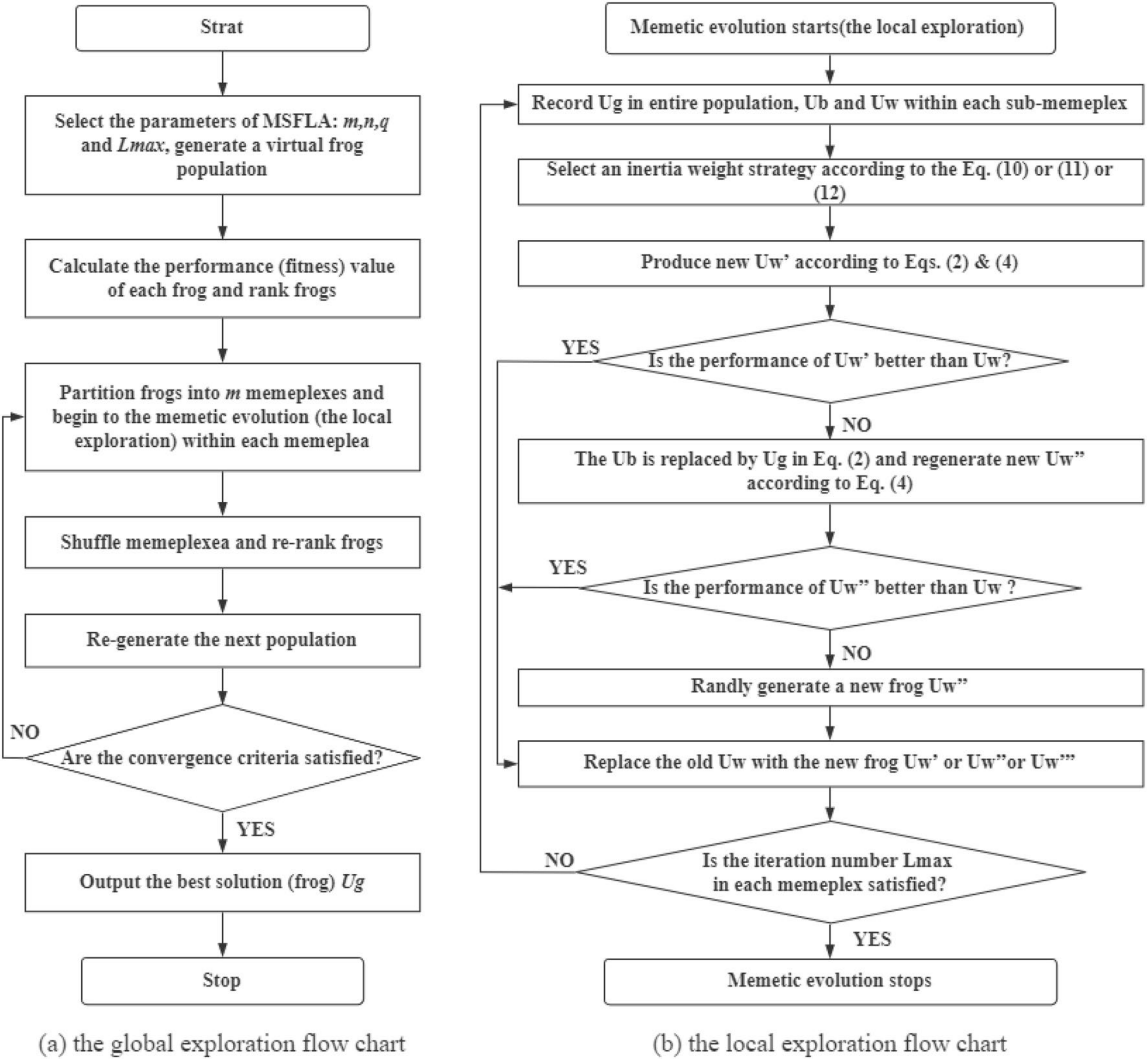
The flow chart of MSFLAs.

Based on the above formula and relevant theories, the flow charts about the global exploration and local exploration (memetic evolution) of 3 MSFLAs are shown in Fig. 3

### Time complexity analysis

For SFLA, the number of individuals in each iteration is unchanged. Assuming that the number of individuals in the algorithm is *m*, the number of global iterations is ${G}_{max}$, the time required for the last update of a single individual in one dimension is *T*, and the spatial dimension of an individual is *D*, the time complexity of SFLA can be obtained as *O (m* × ${G}_{max}$×*T* × *D)*. For MSFLAs, the inertia weight *w* is a fixed value in one iteration, and no repeated calculation is required. Therefore, the effect of introducing *w* on the time *T* required for individual renewal is small and can be ignored. Therefore, the time complexity of MSFLAs s is also *O(m* × ${G}_{max}$×*T* × *D)*. To sum up, the three algorithms of SFLA and MSFLA are the same in terms of time complexity, but MSFLAs obtain better optimization performance due to the optimization and improvement in update strategy.

## Experiment and discussions

In order to evaluate the performance of the MSFLA models, seven well-known benchmark functions are used for testing to assure a reliable comparison^30^. The functions *f*_1_–*f*_3_ and *f*_7_ belong to the unimodal functions which are used to evaluate the exploitation capability of MSFLAs. The *f*_4_–*f*_6_ simulate multi-modal functions to test the exploration performance of MSFLAs. Table 1 shows the basic information of the benchmark functions.

**Table 1:** The benchmark functions.

Name	Function	Range	Dim	Optimal value
Sphere	$$\begin{array}{*{20}c} {f_{1} \left( x \right) = \sum\limits_{i = 1}^{D} {x_{i}^{2} } } & {} \\ \end{array}$$	$$\begin{array}{*{20}c} {} & {\left| {x_{i} } \right|} \\ \end{array} \le 100$$	30	0
Schwefel’s Problem 2.22	$$\begin{array}{*{20}c} {f_{2} \left( x \right) = \sum\limits_{i = 1}^{D} {\left| {x_{i} } \right|} + \prod\limits_{i = 1}^{D} {\left| {x_{i} } \right|} } & {} \\ \end{array}$$	$$\begin{array}{*{20}c} {} & {\left| {x_{i} } \right|} \\ \end{array} \le 10$$	30	0
Schwefel’s Problem 1.2	$$\begin{array}{*{20}c} {f_{3} \left( x \right) = \sum\limits_{i = 1}^{D} {(\sum\limits_{j = 1}^{i} {x_{j} } )^{2} } } & {} \\ \end{array}$$	$$\begin{array}{*{20}c} {} & {\left| {x_{i} } \right|} \\ \end{array} \le 100$$	30	0
Rastrigin’s	$$\begin{array}{*{20}c} {f_{4} \left( x \right) = 10D + \sum\limits_{i = 1}^{D} {\left( {x_{i}^{2} - 10\cos (2\pi \cdot x_{i} )} \right)} } & {} \\ \end{array}$$	$$\begin{array}{*{20}c} {} & {\left| {x_{i} } \right|} \\ \end{array} \le 5.12$$	30	0
Griewangk’s	$$\begin{array}{*{20}c} {f_{5} \left( x \right) = \frac{1}{4000}\sum\limits_{i = 1}^{D} {x_{i}^{2} - \prod\limits_{i = 1}^{D} {\cos \left( {\frac{{x_{i} }}{\sqrt i }} \right) + 1} } } & {} \\ \end{array}$$	$$\begin{array}{*{20}c} {} & {\left| {x_{i} } \right|} \\ \end{array} \le 600$$	30	0
Ackley	$$\begin{array}{*{20}c} {f_{6} \left( x \right) = - 20e^{{ - 0.2\sqrt {\frac{1}{D}\sum\limits_{i = 1}^{D} {x_{i}^{2} } } }} - e^{{\frac{1}{D}\sum\limits_{i = 1}^{D} {\cos 2\pi \cdot x_{i} } }} + 20 + e} & {} \\ \end{array}$$	$$\begin{array}{*{20}c} {} & {\left| {x_{i} } \right|} \\ \end{array} \le 32$$	30	0
Rosenbrock’s Valley	$$\begin{array}{*{20}c} {f_{7} \left( x \right) = \sum\limits_{i = 1}^{D - 1} {\left[ {100(x_{i + 1} - x_{i}^{2} ) + (x_{i} - 1)^{2} } \right]} } & {} \\ \end{array}$$	$$\begin{array}{*{20}c} {} & {\left| {x_{i} } \right|} \\ \end{array} \le 30$$	30	0

All the experiment are performed on a machine with a Core i7 1065G7 CPU, 8-GB memory, and 64 bits Windows 10 operating system. Each algorithm repeats 30 runs independently for eliminating random discrepancy. The algorithm is written based on MATLAB 2019b. For a fair comparison, the base parameters of SFLA and MSFLAs are selected as the same as follows. The number of memeplexes *m* = 25, the number of frog individuals in each memeplex *n* = 25, the number evolved individuals selected from each memeplex *q* = 20, the local iteration number within each memeplex ${L}_{max}$ = 50. The solution accuracy *ɛ*, as one of two stop criteria of algorithms above, are the same in each problem and is less than 1.00E−6 (except *f*_7_ is 30), and another ${G}_{max}$ is equal to 3000 (${G}_{max}$=100*D*). These parameters are set to make a tradeoff between computation time and accuracy. At the same time, the parameters ${\alpha }_{max}$ and ${\alpha }_{min}$ are set to 0.9 and 0.4 in MSFLA-L, *α*_1_ and *α*_2_ are set to 0.9 and 0.2 in MSFLA-Q respectively. The internal parameters of each algorithm are set as shown in Table 2.

**Table 2:** Algorithm parameter setting.

Algorithm	Parameter setting
MSFLA-L	α_max_ = 0.9, α_min_ = 0.4
MSFLA-Q	α_1_ = 0.9, α_2_ = 0.2
ASFLA^19^	*a* = 0.2,${c}_{1}\in [1 2]$
FSFLA^31^	genetic mutation probability ${p}_{m}\in [\mathrm{0.01,0.1}]$
DSFLA^32^	random number $\lambda \in [\mathrm{0,2}]$
BFCEA^33^	the number generated randomly based on Cauchy:$\beta$
LSHADE^34^	*Pbest* = 0.1*, Arcrate* = 2
JADE^34,34^	$p=0.05, c=0.1, crossover probability {\mu }_{CR}=0.5, Cauchy distribution {\mu }_{F}=0.5$
MPA^5^	*P* = 0.5, FADs = 0.2
WOA^4^	Convergence constant $\alpha \in [0 2]$

**Figure 4:**
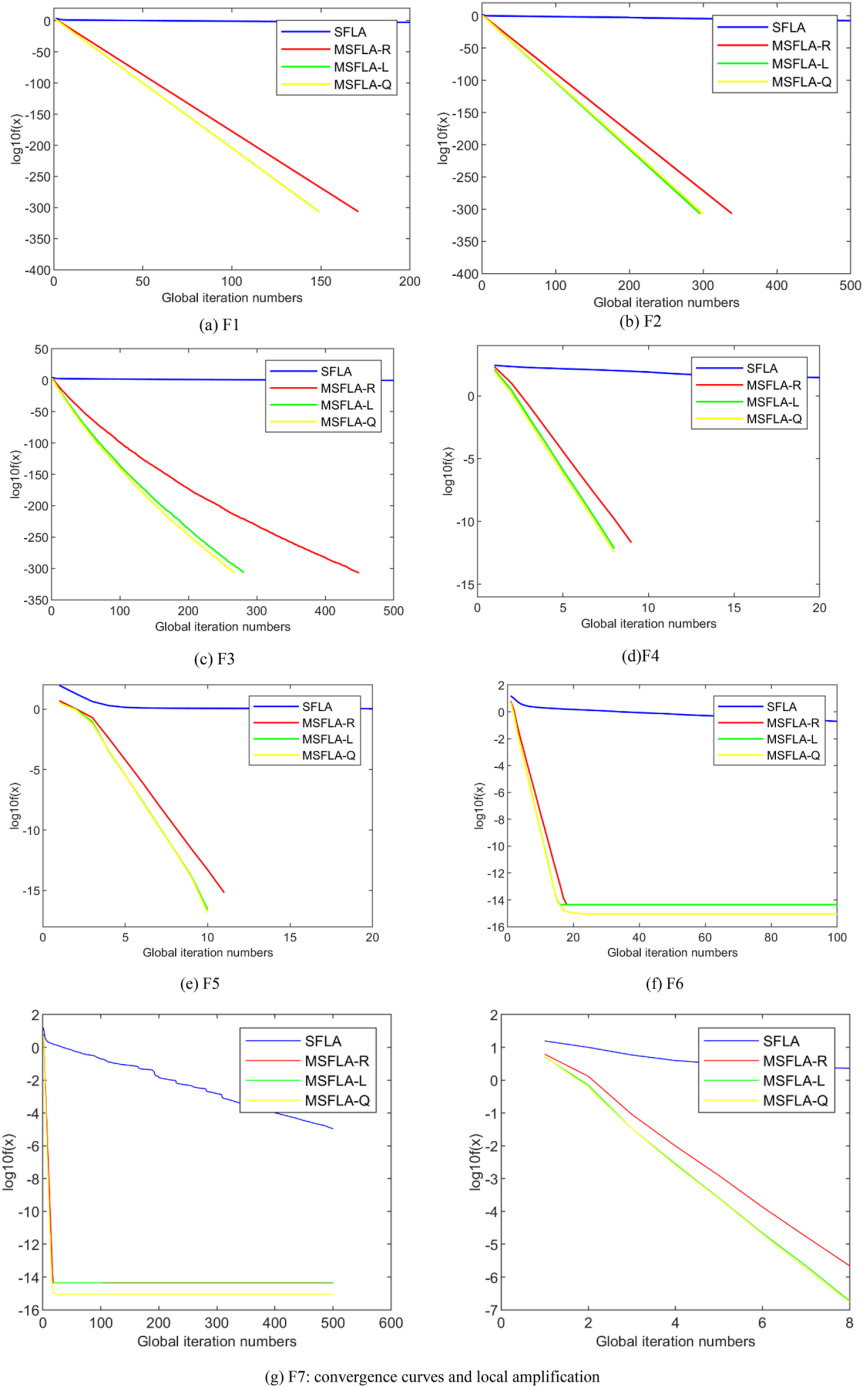
Convergence curves of algorithms on benchmark functions.

The Fig. 4 shows the mean convergence curves (30 independent runs) based on four different algorithms to seven benchmark functions. As can be seen from Fig. 4g, the precision and the convergence speed of the solution based on the three MSFLAs are much better than those of original SFLA. In the solving process of the benchmark function except *f*_6_ and *f*_7_, the values of fitness function using three MSFLAs have been completely converged to the global optimal point when the global iteration number is far less than ${G}_{max}$_*x*_, but the errors of solution based on the original SFLA is relatively large under the same condition. Among of three MSFLAs, the performance of MSFLA-Q and MSFLA-L are similar and both are better than MSFLA-R. There is no notable different in the coordinate values of the tipping points B and C. On the early phase of solution to the f6, the convergence curve based on MSFLA-L coincides with that based on MSFLA-Q, while on the later phase it coincides with that based on MSFLA-R.

To make a comprehensive comparison for the 3 MSFLAs’ performance, the calculation results of 30 independent runs are summarized in Tables 3 and 4. In the two tables, the abbreviation “Std Dev” stands for standard deviation and it can be used to measure the stability of algorithms.

**Table 3:** Speed comparison of solution to 7 benchmark functions based on four algorithms.

Function	Index	Algorithms			
		SFLA	MSFLA-R	MSFLA-L	MSFLA-Q
*f*_1_	Fastest	277	4	4	4
	Slowest	366	5	4	4
	Mean	313.8	4.97	4	4
	Std Dev	22.20	0.18	0	0
*f*_2_	Fastest	287	7	6	5
	Slowest	400	7	6	5
	Mean	336.6	7	**6**	5
	Std Dev	25.13	0	0	0
*f*_3_	Fastest	1882	6	4	4
	Slowest	2658	7	5	5
	Mean	2164.6	6.2	4.93	**4.6**
	Std Dev	205.45	0.4068	0.2537	0.4983
*f*_4_	Fastest	–	5	4	4
	Slowest	–	5	4	4
	Mean	–	5	4	**4**
	Std Dev	–	0	0	0
*f*_5_	Fastest	271	5	4	4
	Slowest	–	5	5	4
	Mean	–	5	4.03	4
	Std Dev	–	0	0.1026	0
*f*_6_	Fastest	468	7	6	6
	Slowest	584	7	6	6
	Mean	497.63	7	6	6
	Std Dev	22.57	0	0	0
*f*_7_	Fastest	98	2	2	2
	Slowest	539	3	2	2
	Mean	207.7	2.3	2	2
	Std Dev	91.70	0.4661	0	0

The symbol ‘–’ indicates that the actual global iteration number is more than 3000 if the accuracy of solution is achieved the specified ɛ.

**Table 4:** The results of the algorithm in test functions in different dimensions.

Func	Algorithm	*D* = 30			*D* = 50			*D* = 100		
		Best	Mean	Std	Best	Mean	Std	Best	Mean	Std
*f*_1_	SFLA	5.61E−53	7.11E−50	1.46E−49	2.30E−32	4.05E−31	5.07E−31	1.73E−16	3.98E−16	1.74E−16
	MSFLA-R	0	0	0	0	0	0	0	0	0
	MSFLA-L	0	0	0	0	0	0	0	0	0
	MSFLA-Q	0	0	0	0	0	0	0	0	0
*f*_2_	SFLA	1.06E−41	1.06E−41	3.33E−40	8.63E−27	8.26E−25	2.42E−24	6.08E−13	2.52E−10	6.56E−10
	MSFLA-R	0	0	0	0	0	0	0	0	0
	MSFLA-L	0	0	0	0	0	0	0	0	0
	MSFLA-Q	0	0	0	0	0	0	0	0	0
*f*_3_	SFLA	2.68E−08	4.27E−07	6.59E−07	1.71E−03	4.22E−.3	2.24E−03	6.76E+00	1.12E+01	2.33E+00
	MSFLA-R	0	0	0	0	0	0	0	0	0
	MSFLA-L	0	0	0	0	0	0	0	0	0
	MSFLA-Q	0	0	0	0	0	0	0	0	0
*f*_4_	SFLA	2.98E+00	6.80E+00	2.40E+00	9.95E+00	1.83E+01	5.60E+00	1.39E+01	2.91E+01	1.17E+01
	MSFLA-R	0	0	0	0	0	0	0	0	0
	MSFLA-L	0	0	0	0	0	0	0	0	0
	MSFLA-Q	0	0	0	0	0	0	0	0	0
*f*_5_	SFLA	9.99E−16	1.90E−02	2.57E−02	3.22E−15	1.26E−02	2.34E−02	4.55E−15	3.70E−03	5.51E−03
	MSFLA-R	0	0	0	0	0	0	0	0	0
	MSFLA-L	0	0	0	0	0	0	0	0	0
	MSFLA-Q	0	0	0	0	0	0	0	0	0
*f*_6_	SFLA	3.87E−12	4.56E−11	4.18E−11	1.28E−11	4.51E−11	3.93E−11	3.84E−09	3.35E−08	8.09E−08
	MSFLA-R	8.88E−16	4.32E−15	6.49E−16	4.44E−15	4.44E−15	0	4.44E−15	4.44E−15	0
	MSFLA-L	8.88E−16	3.73E−15	1.45E−15	4.44E−15	4.44E−15	0	4.44E−15	4.44E−15	0
	MSFLA-Q	8.88E−16	8.88E−16	0	8.88E−16	8.88E−16	0	8.88E−16	8.88E−16	0
*f*_7_	SFLA	1.60E+01	2.55E+01	2.16E−01	3.65E+01	4.57E+01	2.37E+00	8.88E+01	9.89E+01	1.27E+01
	MSFLA-R	2.79E+01	2.77E+01	2.80E+01	4.80E+01	4.82E+01	1.14E−01	9.80E+01	9.83E+01	8.34E−02
	MSFLA-L	2.80E+01	2.82E+01	2.82E+01	4.82E+01	4.84E+01	7.90E−02	9.84E+01	9.85E+01	4.54E−02
	MSFLA-Q	9.04E−02	1.47E−01	1.12E−01	4.81E+01	4.84E+01	1.05E−01	9.85E+01	9.85E+01	3.61E−02

Table 3 shows the calculation speed of four algorithms to those benchmark functions under the same solution precision *ɛ*. Even for the simple unimodal benchmark functions, the original SFLA also needs at least hundreds of operations (global shuffling or iteration numbers) to achieve the required precision. For example, in the process of the solution to the *f*_1_, the fastest speed (the least global iteration number) is 342, while the slowest one is 436, and the mean is 369.4. For some complex or multimodal benchmark functions, they need more global iteration numbers and most of them are even more than 3000. However, these modified SFLAs are used to solve these benchmark functions, the actual global iteration number is often no more than 10. At the same time, the stability of three MSFLAs is far better than the original SFLA.

Table 4 shows the experimental results of four SFLA algorithms in dimension *D* = 30, 50, 100. It can be seen from Table 3 that for *f*_1_–*f*_5_, MSFLAs can reach the theoretical optimal value in three different evaluation indexes and dimensions, while SFLA's convergence accuracy decreases with the increase of dimensions, which indicates the effectiveness of the inertia weight strategy and the suitability of MSFLAs for high-latitude unimodal functions. However, for *f*_6_ and *f*_7_, finding the global optimal solution is quite challenging. The Ackley function f6 is a classical continuous, rotated and non-separable multimodal function. The topological structure feature of f6 is that it is almost everywhere flat on the outer region, but has a non-smooth hole or peak in the middle. *f*_6_ has many local optimal values, which can easily cause the algorithm to stall. The most sought advantage is generally 8.88E-16. With the increase of dimensions, the best and std of MSFLA-Q remain unchanged, and the performance is stable. Secondly, MSFLA-L and MSFLA-R are easy to fall into Local optimization. SFLA performed the worst. The Rosenbrock’s valley function *f*_7_ is a typical ill-conditioned, nonconvex and unimodal function that is difficult to minimize, and there is an obvious correlation between variables. It is a classic optimization problem also known as the banana function. Because this function provides little information for search, it is difficult for many algorithms to identify the search direction when solving, and there is little chance to find the global best. Therefore, this function is also commonly used to evaluate the execution efficiency of optimization algorithms. *f*_7_ is a fixed peak function, when *D* = 30, MSFLAs is better than SFLA, and when *D* = 50, the result is opposite, but when *D* = 100, the results of the four algorithms tend to be similar. This shows that the improved algorithm is less effective in the environment of fixed peak function. In general, for other different types of test functions, MSFLAs is at the bottom of the iteration curve most of the time. The results show that the algorithm has high convergence efficiency, which verifies the effectiveness of the algorithm optimization strategy.

Table 5 shows a comparison of the accuracy of the seven benchmark functions based on other optimization algorithms. The experimental data of these algorithms are derived from references, and the data obtained may vary slightly due to different computer configurations. The data comparison in Table 5 shows that the three MSFLAs proposed in this paper have better robustness and generalization abilities. Even for the function *f*_6_, the precision of solution based on three modified SFLAs are 4–6 orders of magnitude higher than that of the original SFLA and 14–15 orders higher than the three algorithms (ASFLA, FSFLA, and DSFLA), which is basically equivalent to the accuracy of BFCEA algorithm. For the ill-conditioned and nonconvex unimodal function *f*_7_, the accuracy of the improved algorithms is basically the same as that of the original SFLA except the BFCEA and LSHADE algorithm, which shows that they fall into difficulties in solving. Although the LSHADE algorithm adopts a more complex construction form to improve the solution results, there are still many gaps compared with the theoretical value. for nonconvex multimodal and even ill-conditioned functions such as f7, although the convergence accuracy of the four algorithms is almost the same, the number of global iterations when meeting the specified accuracy requirements is significantly reduced and the convergence speed is accelerated. Compared with other intelligent optimization algorithms such as MPA and WOA, three improved SFLAs have obvious advantages in solution accuracy for both unimodal and multi-mode functions. Even the actual results of the three improved SFLAs are exactly the same as the theoretical values (except f6 and f7). This should be attributed to the good ability of local search and global exploration and the potential parallelism of SFLAs algorithm themselves.

**Table 5:** Precision comparison of solution to 7 benchmark functions based other optimization algorithms.

Func	Index	Algorithms							
		ASFLA	FSFLA	DSFLA	BFCEA	LSHADE	JADE	WOA	MPA
*f*_1_	Best	4.24E−220	9.84E−62	6.44E−05	0	–	–	–	1.17E−52
	Mean	5.21E−214	4.06E−57	1.23E−02	5.89E−315	1.12E−90	0	1.41E−30	5.84E−50
	Std	0	1.19E−56	2.51E−02	0	6.44E−90	0	4.91E−30	6.46E−50
*f*_2_	Best	1.44E−121	1.26E−38	7.83E−02	4.04E−204	–	–	–	4.27E−30
	Mean	5.20E−120	1.26E−37	1.60E+00	1.39E−196	2.09E−42	2.09E−42	1.06E−21	5.43E−28
	Std	6.99E−120	2.05E−37	1.84E+00	0	1.03E−41	1.03E−41	2.39E−21	8.81E−28
*f*_3_	Best	8.00E−08	4.47E−04	6.61E+02	0	–	–	–	1.27E−23
	Mean	5.95E−07	2.41E−03	1.40E+03	3.88E−302	3.85E−81	3.58E−49	5.39E−07	2.50E−12
	Std	6.70E−07	1.55E−03	7.53E+02	0	1.74E−80	7.53E−49	2.93E−06	3.71E−12
*f*_4_	Best	1.49E+01	1.09E+01	1.30E+01	0	–	–	–	0
	Mean	2.51E+01	1.41E+01	1.70E+01	2.47E−13	1.74E−16	0	0	0
	Std	6.73E+00	3.17E+00	3.76E+00	7.81E−13	6.35W-16	0	0	0
*f*_5_	Best	0	0	7.43E−04	0	–	–	–	0
	Mean	3.20E−03	1.64E−02	4.47E−02	1.24E−15	0	1.55E−03	2.89E−04	0
	Std	5.54E−03	2.32E−02	2.92E−02	5.52E−15	0	3.81E−03	1.59E−03	0
*f*_6_	Best	3.82E−01	1.55E+00	1.49E+00	7.88E−16	–	–	–	8.88E−16
	Mean	2.59E+00	1.47E+01	1.18E+01	8.78E−16	4.00E−15	4.76E−15	7.40E+00	3.85E−15
	Std	6.12E+00	8.53E+00	9.64E+00	3.16E−17	2.37E−30	1.46E−15	9.90E+00	1.35E−15
*f*_7_	Best	3.21E−05	8.51E−03	2.93E+01	1.88E−05	–	–	–	2.29E+01
	Mean	4.28E+00	1.16E+01	7.22E+01	5.16E−05	1.40E−25	1.85E+01	2.79E+01	2.40E+01
	Std	1.51E+01	2.15E+01	2.96E+01	2.49E−05	9.70E−25	1.01E+01	7.64E−01	5.38E−01

The Wilcoxon Signed-Rank test is the most popular non-parametric test in statics and it can be applied to determine if two sets of solutions (population) are different statistically significant or not^35^. Each set of pairs in both populations are compared to calculate and analyze their numerical differences based on this method. In short, the Wilcoxon Signed-Rank test returns a numerical result called p-value. The p-value determines the significance level of two different algorithms. An algorithm is statistically significant if and only if it results in the p-value less than 5%. The *p*-values in Table 6 also show that this superiority is statistically significant since the all *p*-values are much less than 5%, which further reflect the robustness of the proposed MSFLA algorithms.

**Table 6:** Thirty times *P*-values of Wilcoxon Signed-Rank test.

Function	Algorithms		
	MSFLA-R	MSFLA-L	MSFLA-Q
*f*_1_	1.21E−12	1.21E−12	1.21E−12
*f*_2_	1.21E−12	1.21E−12	1.21E−12
*f*_3_	1.21E−12	1.21E−12	1.21E−12
*f*_4_	1.21E−12	1.21E−12	1.21E−12
*f*_5_	1.21E−12	1.21E−12	1.21E−12
*f*_6_	1.21E−12	1.21E−12	1.21E−12
*f*_7_	2.32E−06	2.32E−06	1.86E−06

In general, the foregoing simulation results reveal that three proposed algorithms with different inertia weight strategy are superior over original SFLA in terms of adaptability, stability, and the rapid global search ability.

## Engineering design problems

To verify the feasibility of MSFLAs in solving constrained optimization problems in engineering design, three MSFLAs and the SFLA algorithm are applied to the case for the optimal design of tension/compression spring and cantilever beam. They are both multi-constrained and single-objective functions. The algorithm parameters and population size are constant, and the maximum number of iterations is 1000. Each algorithm was run 30 times independently.

### Tension/compression spring design problem

**Figure 5:**
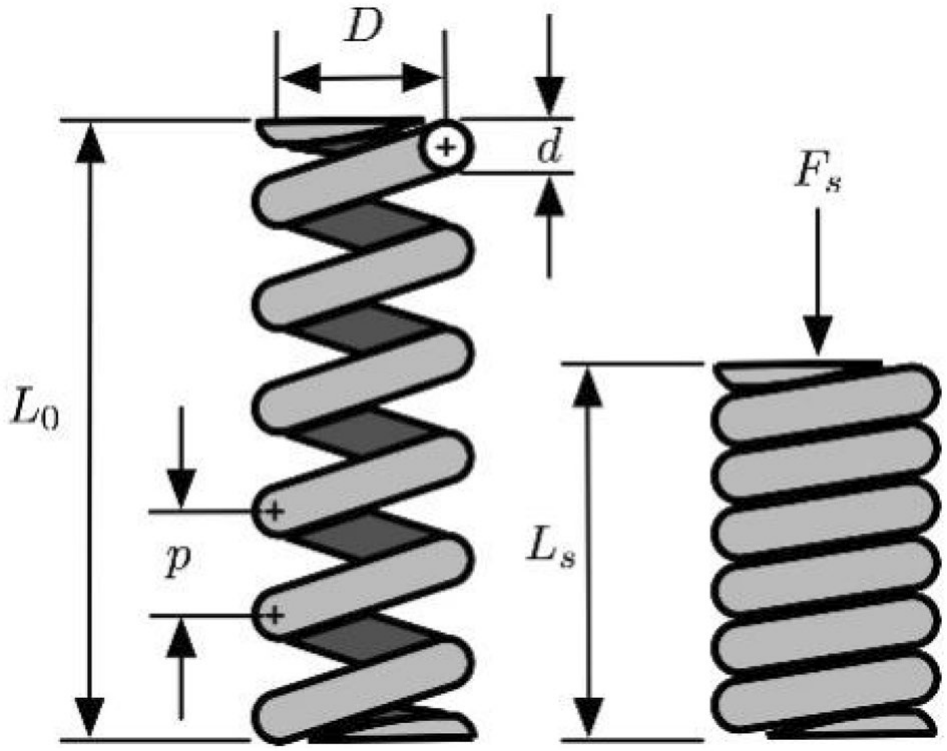
Tension/compression spring design problem.

The goal of tension/compression spring optimization is to minimize the weight of the spring in Fig. 5. The variable is the average diameter of the spring coil *d* (*x*_1_/*cm*), the diameter of the spring wire *D* (*x*_2_/*cm*) and the effective number of coils of the spring *N* (*x*_3_). The constraint conditions are the minimum deflection (*g*_1_), shear stress (*g*_2_), impact frequency (*g*_3_) and outer diameter limit (*g*_4_) ^36^. The specific mathematical model is as follows:

Function:

$$\min f(x) = (x_{3} + 2)x_{2} x_{1}^{2}$$

Subject to:

$$g_{1} (x) = 1 - \frac{{x_{2}^{3} x_{3} }}{{71785x_{1}^{4} }} \le 0;$$

$$g_{2} (x) = \frac{{4x_{2}^{2} - x_{1} x_{2} }}{{12566\left( {x_{2} x_{1}^{3} - x_{1}^{4} } \right)}} + \frac{1}{{5108x_{1}^{2} }} - 1 \le 0;$$

$$g_{3} (x) = 1 - \frac{{140.45x_{1} }}{{x_{2}^{2} x_{3} }} \le 0;$$

$$g_{4} (x) = \frac{{x_{1} + x_{2} }}{1.5} - 1 \le 0;$$

$$0.05 \le x_{1} \le 2,0.25 \le x_{2} \le 1.3,2 \le x_{3} \le 15$$

Table 7 records the comparison experiments of the optimal values of the tension/compression spring design problem. The data in the table are average values. The results of all three MSFLAs are better than the basic SFLA, which indicates that MSFLAs have better optimization accuracy in solving this problem (Supplementry information).

**Table 7:** Comparison of results for tension/compression spring design problem.

Algorithm	Optimal values for variables			Optimal weight
	*x*_1_	*x*_2_	*x*_*3*_	
SFLA	0.06113	0.59251	4.97670	0.01355
MSFLA-R	0.05734	0.50712	6.05552	0.01294
MSFLA-L	0.05865	0.54109	5.60096	0.01325
MSFLA-Q	0.05877	0.54162	5.63351	0.01326

### Cantilever beam design problem

**Figure 6:**
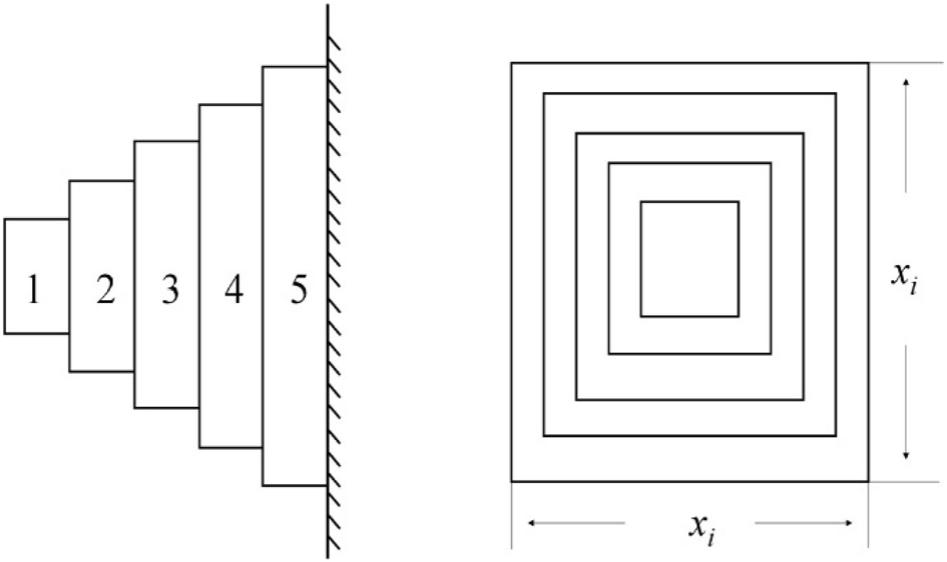
Cantilever beam design problem.

The cantilever beam design is shown in Fig. 6, which consists of five hollow members. The objective is to reduce the weight of the cantilever beam. The variable is the cross-sectional width *x*_*i*_ (*i* = 1,2,…, 5/*cm*). The constraint is the deflection of the cantilever beam^37^. The mathematical model is as follows:

Function:

$$\min f(x) = 0.0624(x_{1} + x_{2} + x_{3} + x_{4} x_{5} )$$

Subject to:

$$g_{1} (x) = \frac{61}{{x_{1}^{3} }} + \frac{37}{{x_{2}^{3} }} + \frac{19}{{x_{3}^{3} }} + \frac{7}{{x_{4}^{3} }} + \frac{1}{{x_{5}^{3} }} - 1 \le 0;$$

$$0.01 \le x_{i} \le 100,i = 1,2,3,4,5$$

It can be seen from Table 8 that MSFLAs provide the best value in the cantilever beam design problem, and the variable solutions of MSFLAs are reduced sequentially, while the gap between the variable solutions of SFLA is too small for practical design difficulties. The result shows that the search performance of MSFLAs is more powerful than the original algorithm.

**Table 8:** A comparison of results for the cantilever beam design problem.

Algorithm	Optimal values for variables					Optimal weight
	${x}_{1}$	${x}_{2}$	${x}_{3}$	${x}_{4}$	${x}_{5}$	
SFLA	5.18404	5.02278	4.75330	4.60001	4.60786	1.42472
MSFLA-R	6.00314	5.34248	4.50443	3.52910	2.13445	1.34112
MSFLA-L	6.05465	5.28922	4.55112	3.52522	2.16234	1.34675
MSFLA-Q	6.03504	5.41168	4.52976	3.53117	2.16483	1.34350

## Conclusions

In this paper, a modified shuffled frog leaping algorithm (MSFLA) has been developed by introducing the inertia weight. According to different inertia weight strategies, three improved SFLAs are formed. The global convergence of the original SFLA has been proved through establishing the Markov chain model, as long as the global iteration (shuffling) number is large enough in the literature^38^. In the proposed MSFLAs, the update strategies with inertia weight only appropriately increase the diversity of candidate solutions (frogs) to obtain the optimal solution as earlier as possible. Essentially, the computational complexity about the two classes of algorithms, namely SFLA and MSFLAs, are the same, i.e. Therefore, the global convergence of three modified SFLAs can be ensured. The results of seven typical testing functions show that the proposed MSFLAs have the excellent global optimization ability, the local exploration ability and the generalization abilities. Furthermore, it can effectively improve the solution precision of complex functions in a high-dimensional space, and accelerate the convergence speed. Among of them, the performance of MSFLA-Q is the best and it means that the nonlinear time-varying inertia weight strategy is the most effective.

The present work has some limitations. Firstly, the scale of the simulation functions applied in this paper is relatively small, some large-scale and higher-dimensional studies should test our improved algorithm. Second, in terms of solution accuracy, the advantage of the 3 MSFLAs algorithm for seriously ill conditioned nonconvex functions is not obvious, and the improvement needs to be further studied. Finally, further research can focus on verifying the modified SFLA with inertia weight in terms of the practice optimization problems in industrial production and other applications.

### Supplementary Information

Supplementary Information.

## Data Availability

All data generated or analysed during this study are included in this published article [and its supplementary information files.
